# Case Report: Esophageal squamous cell carcinoma in a 13-year-old boy with a history of esophageal atresia with tracheoesophageal fistula

**DOI:** 10.3389/fped.2024.1438242

**Published:** 2024-10-11

**Authors:** B. Bernar, C. Mayerhofer, T. Fuchs, G. Schweigmann, E. Gassner, R. Crazzolara, B. Hetzer, U. Klingkowski, A. Zschocke, G. Cortina

**Affiliations:** ^1^ Department of Anesthesia and Intensive Care Medicine, Medical University of Innsbruck, Innsbruck, Austria; ^2^ Department of Pediatrics I, Medical University of Innsbruck, Innsbruck, Austria; ^3^ Department of Pediatrics, Regional Hospital Reutte, Reutte, Austria; ^4^ Department of Pediatrics III, Medical University of Innsbruck, Innsbruck, Austria; ^5^ Department of Radiology, Pediatric Radiology, Medical University of Innsbruck, Innsbruck, Austria; ^6^ Department of Radiology, Medical University of Innsbruck, Innsbruck, Austria

**Keywords:** esophageal atresia, ESCC (esophageal squamous cell carcinoma), child—age, esophageal repair, cancer

## Abstract

In adults, esophageal cancers are a global health concern. Esophageal squamous cell carcinoma (ESCC) accounts for approximately 90% of esophageal carcinomas. The prognosis of esophageal cancers remains dismal, with a five-year survival rate below 20%. It typically affects older patients, and for now, ESCC after esophageal atresia has not been reported in patients younger than 18 years. We present an exceptional case of an ESCC in a 13-year-old boy with a history of esophageal atresia and corrective surgery in infancy. After the surgery the patient was lost to surgical follow up for over ten years and then presented to our emergency department with respiratory distress requiring antibiotic therapy and supplemental oxygen. Radiologic imaging revealed a volume reduction of the right lung with bronchiectasis, as well as esophageal stenosis at the level of the previous anastomosis, with an adjacent abscess in the right lung. These changes may have arisen due to a chronic fistula from the esophagus to the right lung. Initial interventional therapy with a stent implantation had no lasting success and, in an effort to prevent further aspiration into the right lung, a cervical esophagus stoma was established, and the patient received prolonged antibiotic treatment. However, a thoracic CT scan performed 4 months later revealed a large, retrospectively progressive prevertebral mass originating from the distal portion of the esophagus below the stenosis, compressing the trachea and the right main bronchus. The patient's condition rapidly worsened and he developed respiratory failure, requiring veno-venous extracorporeal membrane oxygenation. Unfortunately, an endoscopic biopsy revealed an advanced ESCC. With no rational treatment options available, we changed the goals of care to a palliative setting. The key message of this case is that in adolescents with chronic infections, an abscess can potentially mask a malignant transformation. Therefore, in adolescents, with an history of corrective surgery for esophageal atresia and chronic complications, consideration should also be given to the possibility of squamous cell carcinoma of the esophagus.

## Introduction

In Western countries, esophageal adenocarcinoma has emerged as the predominant subtype of esophageal malignancies during the past three decades (1). However, approximately 90% of esophageal carcinomas worldwide are still classified as squamous cell carcinomas (2). Esophageal squamous cell carcinoma (ESCC) commonly arises from chronic inflammation resulting from environmental and lifestyle factors. There is a significant gender-based association with esophageal carcinoma (M:F 4.4:1) (2), particularly due to risk factors such as smoking and alcohol consumption (3–5). The overall survival is poor, with a five-year survival rate of less than 20% (2, 4, 6). However, it improves for early-stage cancers, underscoring the importance of early diagnosis. Generally, the risk of ESCC increases with age, with an average onset occurring around 60 to 70 years (7–9). However, in patients after esophageal repair, malignant transformation can occur earlier.

In 1987, a case of squamous cell carcinoma was documented for the first time in a 45 years old women following antethoracic skin tube esophageal conduit surgery (10). Similarly, in 1989, esophageal adenocarcinoma was reported in a 20-year-old woman with a history of esophageal atresia (11). Esophageal atresia (EA) has a prevalence of 1:2,500 to 1:4,000 life birth (12) and is a severe, life-threatening, congenital malformation characterized by the complete discontinuity of the esophagus. Today, longterm survival rates for patients with successfully corrected EA are nearly 100% (13, 14). The long-term survival of patients, however, has also brought new challenges, particularly through the emergence of long-term complications such as tracheomalacia, anastomotic stricture, new fistulae, and gastroesophageal reflux (15, 16). These complications have the potential to result in chronic aspiration, recurrent pneumonia, dysphagia, and inflammation, establishing a cycle that could ultimately culminate in the earlier described malignant transformation (15).

According to current literature, malignant transformation following esophageal atresia correction is typically reported after decades, mostly occurring in the late 30s or thereafter (9, 17). So far, 18 patients have been reported, 12 with ESCC, 5 with EAC and one with pulmonary squamous cell carcinoma. In 15/17 complications following initial repair have been reported (i.e., stricture, fistulae, GERD) and in 8/17 at least one risk factor (smoking, alcohol consumption or GERD) was reported. For now, the youngest patients were 20 and 19 years old at the time of diagnosis. The mean age for ESCC patients was 39.3, and for EAC patients, it was 34.0 years. None of the previously reported 17 cases was younger than 18 years (9–11, 17–22). However, we encountered an exceptionally early case which is presented here.

## Case presentation

### Initial presentation

We present the case of a 13-year-old Caucasian boy who presented to our emergency department in the summer of 2022 in respiratory distress with an oxygen saturation at presentation of less than 80% in room air. During initial assessment, the mother reported that her son had been facing breathing difficulties during physical exercise for some time. Upon further inquiry, the mother revealed that her son was born with esophageal atresia. Although the corrective surgery had various complications, the boy was described as being generally healthy since then. According to his mother, his regular oxygen saturation at home typically ranged between 80% and 90%. Recent start of recurrent episodes of cyanosis while walking has led to the regular saturation controls at home, and after deterioration to the actual hospital presentation. Furthermore, the mother reported that he experienced three episodes of hemoptysis. The boy, himself reported, that he has only mild respiratory distress during physical activity.

**Figure 1:**
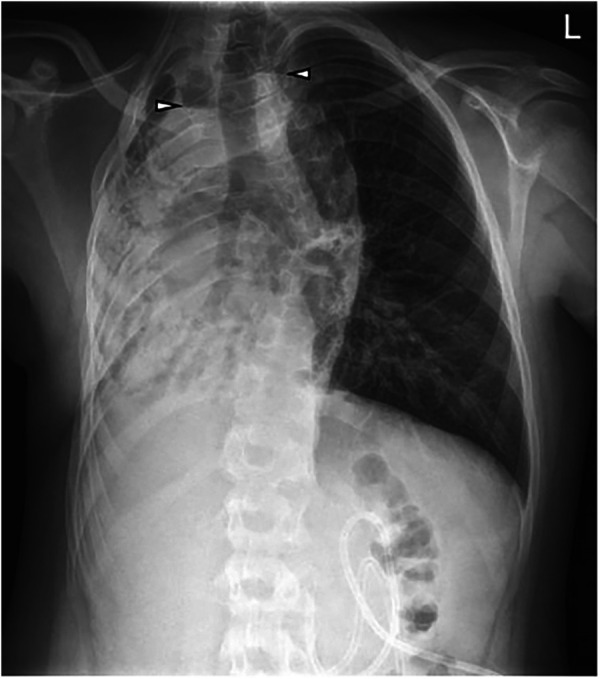
Initial chest x-ray at the age of 13 years 6 months: G-tube in the stomach. Volume loss and opacification of the right lung with focal air-filled bronchiectasis. Two areas with air-fluid levels in the upper mediastinum and right lung (arrowheads). Compensatory hyperinflated left lung.

**Figure 2:**
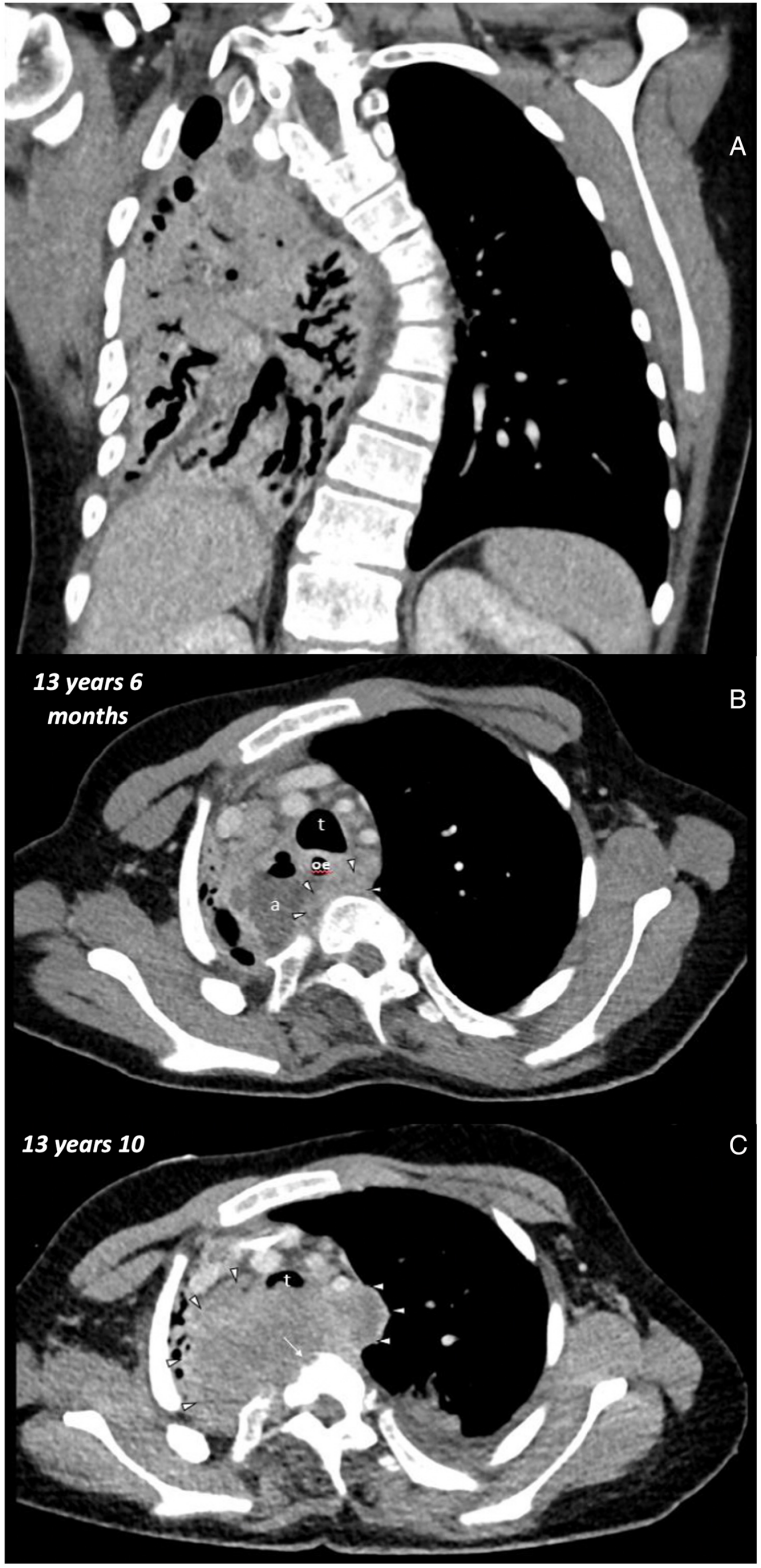
Initial chest CT at the age of 13 years 6 months: **(A)** coronal soft tissue window thorax: volume loss of the right lung with bronchiectasis. Left convex scoliosis. **(B)** Axial soft tissue window upper thorax: trachea (t), esophagus (oe), abscess (a), soft tissue mass (arrowheads). Second chest CT at the age of 13 Years 10 months: **(C)** axial soft tissue window upper thorax: trachea (t), soft tissue mass (arrows heads), bony errosions (long arrow).

During examination, a brown-black discolored gastrostomy tube (G-tube) became obvious, and the mother clarified that the boy still receives nutrition through this G-tube, nutritional status was not reduced. Auscultation of the lung revealed the absence of breath sounds on the right side. The imaging studies (chest x-ray and chest CT) revealed volume loss in the right lung with bronchiectasis, as well as esophageal stenosis at the level of the previous anastomosis, with an adjacent abscess in the right lung (Figures 1, 2). These changes may have arisen due to a chronic fistula from the esophagus to the right lung.

### Earlier childhood

**Figure 3:**
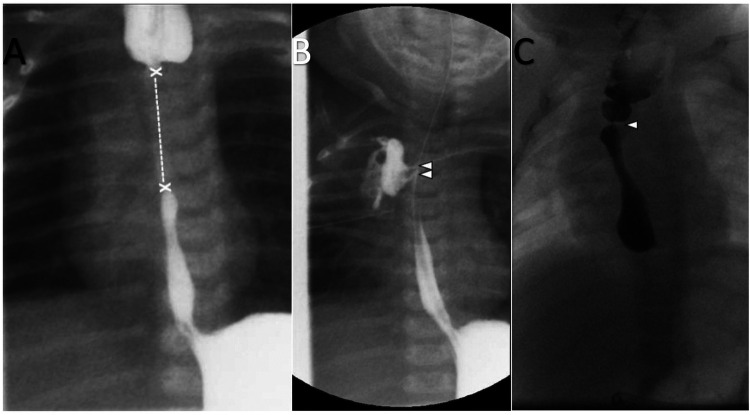
**(A)** Fluoroscopy at the age of 6 weeks: application of contrast medium in the esophageal tube and G-tube: long gap (x—x) of the esophageal atresia. **(B)** Fluoroscopy at the age of 10 weeks: Post end-to-end anastomosis of esophagus atresia. Nasogastric tube. Application of contrast medium in G-tube. Contrast leakage from the esophagus to the right lung/pleura (arrowheads). **(C)** Fluoroscopy at the age of 6 months: Oral administration of contrast medium. Dilated proximal esophagus. Focal esophageal stenosis without leakage (arrowhead).

Following the imaging, the boy was admitted for further treatment. A comprehensive review of the boy's medical records revealed that the presumed G-tube was not a gastrostomy but a jejunal tube. Its last replacement had occurred when the boy was one and a half years old. As a neonate, the patient underwent surgical intervention due to esophageal atresia Vogt Type IIIb with a long gap (Figure 3). The tracheoesophageal fistula (TEF) was ligated on the second day of life. A primary end-to-end anastomosis was performed two months after birth; however, an anastomotic dehiscence, with leakage to the right lung and a short thin stenosis, occurred two weeks later and was successfully oversewn. Afterwards, regular dilatation procedures would have been required, however the parents reported recurrent respiratory infections, which resulted in frequent delays of the procedures.

At the age of two years, the surgeons conducted their last assessment before the boy was lost to surgical follow-up. During this last evaluation, the surgeons recommended a stent implantation to stabilize a stenosis at the anastomosis site. From that point until his presentation to our emergency department, the boy was mainly monitored by the family doctor. The family doctor assumed that the boy was still routinely seen by the surgeons. The parents reported that the boy frequently required antibiotic therapy for respiratory infections such as pneumonia, with the last treatment taking place in fall 2021. Three years prior to his current presentation, the boy was admitted to a peripheral hospital due to infectious laryngotracheitis. According to the parents’ description, oxygen saturation had been low for many years. Concerns regarding recurring episodes of cyanosis, chronic desaturation, and hemoptysis have emerged in recent months.

### Diagnostic assessment

In addition to the initial Chest x-ray (Figure 1) and CT (Figure 2), a blood work revealed indications of an infection, with a white blood cell count (WBC) of 19.7 G/L (reference: 4–10 G/L), and a C-reactive protein (CRP) level of 16.71 mg/dl (reference: <0.5 mg/dl). Additionally, the presence of *Escherichia coli* and *Klebsiella oxytoca* were detected in a sputum sample. Vital signs recorded at admission were as follows: transcutaneous oxygen saturation of 86% in room air, a respiratory rate of 28 per minute, heart rate of 150 per minute, and a blood pressure of 113/77 mmHg.

### Initial therapeutic decisions

As we diagnosed a respiratory infection and an abscess between the esophagus and trachea (CT evidence), an antibiotic therapy with amoxicillin and clavulanic acid was initiated. We suspected a fistula between esophagus and trachea as inflammation-trigger. To normalize oxygen saturation supplemental oxygen was administered. After consultation with our thoracic surgeons, a decision was made to transfer the patient to a specialized pediatric surgical clinic.

### Further therapy

At the specialized pediatric surgical center, a stent was introduced into the esophagus and for a brief period, the patient came off supplemental oxygen, followed by another episode of respiratory deterioration and need for oxygen supplementation, this time caused by an erosion of the trachea in the region of the stent. To address this complication, a cervical esophagus stoma was performed, with the other end of the esophagus being blindly detached. Again, antibiotic therapy was intensified and additionally, non-invasive ventilation (NIV) became necessary. At the end, the patient was discharged home with NIV. Due to the fact, that the initial CT images showed evidence for an abscess, an oncological etiology was at this timepoint not suspected, explaining why no biopsies were obtained at this stage.

### Outcome

In the autumn of 2022, the boy returned to our emergency room, requiring intensified non-invasive ventilation and was readmitted for further assessment. He already was on oral antibiotics (Cefadroxil and Ciprofloxacin) and antibiotic inhalations (Colistin/Tobramycin).

On re-admission, the patient showed signs of respiratory distress with decreased oxygen saturation (92%), tachypnoea (44 breaths per minute), tachycardia (150 beats per minute) and hypertension (142/96 mmHg). Laboratory results showed a leukocytosis (WBC of 15.1 G/L) and blood gas analysis showed elevated CO_2_ partial pressure of 62.7 mmHg. The key findings in the chest x-ray include impaired ventilation in the right upper and lower fields and an obscured infiltrate in the lower right field. In contrast to the initial chest x-ray the right lung now appeared to be slightly better ventilated. In addition to the already known *Escherichia coli*, *Achromobacter xylosoxidans* was cultured in sputum samples. A targeted intravenous antibiotic therapy with Piperacillin and Tazobactam was initiated. Additionally, due to respiratory distress, intravenous administration of prednisolone was initiated, and inhalation therapy was intensified with ipratropium.

**Figure 4:**
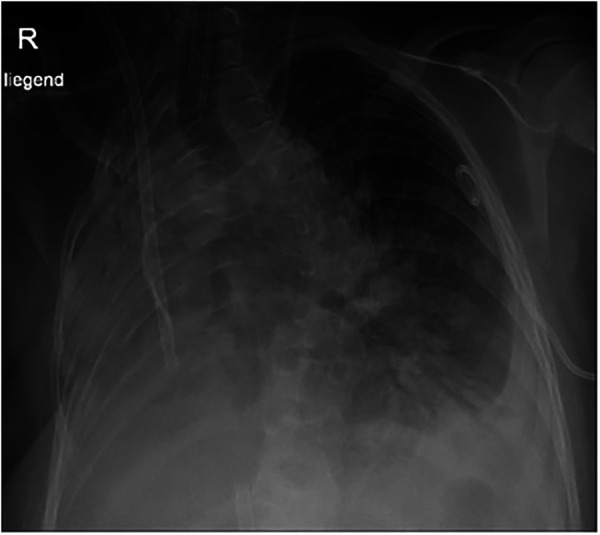
Chest x-ray at the age of 13 years 10 months: tracheal tube, ECMO tubes, left thoracic pigtail drain. Volume loss and opacification of the right lung. Basal pleural effusion on the left.

However, respiratory distress continued to worsen over the following days, and the boy required endotracheal intubation and invasive mechanical ventilation. Following intubation, bradycardia occurred, leading to the necessity of a short episode of cardiopulmonary resuscitation (CPR) with rapid return of spontaneous circulation (ROSC). Subsequently, the boy developed hypoxic respiratory failure and was evaluated for extracorporeal membrane oxygenation (ECMO). Veno-venous ECMO was placed via the right internal jugular vein and the right femoral vein (Figure 4) and rapidly improved oxygenation and CO_2_ removal. To clarify the situation, a second thoracic CT scan (Figure 2) was performed. It revealed a large, retrospectively progressive in size, prevertebral mass originating from the distal esophageal segment below the stenosis, compressing the trachea and the right main bronchus.

At this point it was evident that the right lung could not be saved medically and required a surgical intervention. Options were discussed with our thoracic surgeons and given the complexity of the condition and the unknown etiology of the large mass, we transferred the boy with ECMO to the national lung transplant center in Vienna for further surgical evaluation. An endoscopic biopsy of the mass revealed an invasive, moderately differentiated, keratinized squamous cell carcinoma of the distal esophagus. Due to the invasive growth causing destruction of the esophagus, erosion of the trachea, involvement of the aorta, vertebral bodies, and the lungs, unfortunately, no rational treatment options were available. At the family's request, the boy was transferred back to Innsbruck, where ECMO therapy was terminated. An autopsy was conducted, and histologic evaluation confirmed an ESCC and classified the tumor expansion as pT4b, pN2, L1, V0, Pn0.

## Discussion

This tragic case emphasizes the critical importance of routine surgical or gastroenterological follow-up examinations, which could have played a pivotal role in early detection and intervention. Timely surgical intervention for the anastomotic stenosis, identified in early childhood of our patient, could likely have prevented the development of chronic inflammation and, ultimately, malignant transformation.

In our case, the parents reported feeling traumatized by multiple interventions and admissions to the intensive care unit during infancy. This led them to avoid seeking higher levels of healthcare services for many years. This highlights the importance of offering psychological support to families whose children had a challenging start in life (i.e., involving extended stays in intensive care or multiple surgeries).

Trust, but verify: Controls following corrective surgeries for esophageal atresia (but also other surgeries in early life) should adhere to a fixed protocol and be documented. This includes ensuring that treating physicians are automatically reminded of appointments if patients fail to attend. The family doctor was unaware that the boy had not received surgical follow-ups for years and primarily focused on his own area of expertise. This is certainly also due to the fact that general practitioners must care for an increasing number of patients, resulting in less time available for each patient. Exacerbating this problem, various hospitals employ different documentation systems, often without compatibility, and primary care physicians frequently lack access to either hospital documentation systems or those used by other physicians. However, in complex cases such as those involving a history of esophageal atresia, preterm birth, or metabolic disease, facilitating communication and organizing follow-ups are crucial elements that should be assisted by digital tools and reminders. Furthermore, this responsibility should not be transmitted onto the family doctor but rather should be assumed by specialized centers.

All these measures should aim to minimize the number of patients that are lost to follow-up. This case underscores the potential for tragic outcomes when multiple oversight mechanisms fail, involving not only the parents and doctors, but also the family surrounding and educational staff.

The aforementioned factors all contributed to the development of this tragic outcome. However, the most crucial lesson remained hidden at first and only emerged through retrospective evaluation. Current literature has documented 17 patients with ESCC or EAC following previous esophageal atresia (9–11, 17–23), but none of these patients were minors. So far, only 3 patients younger than 25 years have been reported, aged 19 (20), 20 (11), and 22 (9) years old. Two of them presented with metastatic disease at the time of diagnosis, and both had an unfavorable outcome (9, 20). The average age of all 18 patients (including the one presented here) is 36.7 years. A large meta-analysis indicated that 11% of patients with esophageal carcinomas (*n* = 13,331) were younger than 50 years, but none were younger than 18 years (24).

In the initial CT scans, the existing abscess was clearly identified. However, an esophageal carcinoma obscured by the abscess was not yet considered due to the patient's young age and the location. Only when the initial antibiotic therapy and surgical interventions (stent placement) did not yield the desired results, doubts arose, and further imaging was performed. The subsequent CT images revealed the rapid growth of the squamous cell carcinoma, which was retrospectively understood to have been initially obscured by the abscess. Reflective analysis of the CT images showed a soft tissue mass marginal to the abscess already aged 13 years and 6 months. However, it is only in the later images at 13 years and 10 months that bone erosions, tissue infiltration, and size increase became apparent, leading to a strong suspicion of an oncological origin at this point.

Thus, the key message of this case is that in adolescents with chronic infections, an abscess can potentially mask a malignant transformation. Therefore, in adolescents, with an history of corrective surgery for esophageal atresia and chronic complications, consideration should also be given to the possibility of squamous cell carcinoma of the esophagus.

## Data Availability

The original contributions presented in the study are included in the article/Supplementary Material, further inquiries can be directed to the corresponding author.
