# Supplementary material for: Case Report: Esophageal squamous cell carcinoma in a 13-year-old boy with a history of esophageal atresia with tracheoesophageal fistula
Source: Front Pediatr. 2024 Oct 11;12:1438242. doi: 10.3389/fped.2024.1438242 (PMC11502401; doi:10.3389/fped.2024.1438242)
Supplement: Supplementary file 1 [file Image1.pdf]

Earlier childhood

- Esophageal atresia Typ Vogt IIIb
- Tracheo-esophageal fistula ligated on the second day of life
- At 6 weeks of life fluroscopy (Figure 1)
- At 8 weeks of life primary end-to-end anastomosis
- At 10 weeks of life leakage and thin stenosis (Figure 1)
- Regular dilation procedures due to stenosis (Figure 1)
- At two years of life lost to surgical follow-up

Later Childhood

- Regular respiratory tract infections an pneumoniae
- Reported episode with cyanosis and respiratory distress

Aged 13 years

- At 13 years 6 months: Respiratory distress with cyanosis and episodes of hemoptysis
  - X-Ray and CT-scan (Figures 2-3): Volume loss of the right lung with bronchiectasis, abscess and a soft tissue mass
  - Diagnosis: Pneumonia with mediastinal abscess
  - Antibiotic therapy and surgical intervention, NIV became necessary
- At 13 years 10 months: Deterioration with respiratory distress and respiratory failure
  - Hypoxic respiratory failure and need of extracorporeal membrane oxygenation (ECMO)
  - CT-scan (Figures 3-4): large mediastinal mass with bone errosions
  - Bronchoscopy: ESCC
  - No rational treatment options were available
  - ECMO therapy was terminated

Postmortem

- Histologic evaluation confirmed an ESCC and classified the tumor expansion as pT4b, pN2, L1, V0, Pn0.
